# Fusion of Smartphone Motion Sensors for Physical Activity Recognition

**DOI:** 10.3390/s140610146

**Published:** 2014-06-10

**Authors:** Muhammad Shoaib, Stephan Bosch, Ozlem Durmaz Incel, Hans Scholten, Paul J. M. Havinga

**Affiliations:** 1 Pervasive Systems Group, Department of Computer Science, Zilverling Building, PO-Box 217, 7500 AE Enschede, The Netherlands; E-Mails: stephan@inertia-technology.com (S.B.); hans.scholten@utwente.nl (H.S.); p.j.m.havinga@utwente.nl (P.J.M.H.); 2 Department of Computer Engineering, Galatasaray University, Ortakoy, Istanbul 34349, Turkey; E-Mail: odurmaz@gmail.com

**Keywords:** accelerometer, activity recognition, assisted living, gyroscope, health monitoring, magnetometer, sensor fusion, smartphone sensors, wellbeing applications

## Abstract

For physical activity recognition, smartphone sensors, such as an accelerometer and a gyroscope, are being utilized in many research studies. So far, particularly, the accelerometer has been extensively studied. In a few recent studies, a combination of a gyroscope, a magnetometer (in a supporting role) and an accelerometer (in a lead role) has been used with the aim to improve the recognition performance. How and when are various motion sensors, which are available on a smartphone, best used for better recognition performance, either individually or in combination? This is yet to be explored. In order to investigate this question, in this paper, we explore how these various motion sensors behave in different situations in the activity recognition process. For this purpose, we designed a data collection experiment where ten participants performed seven different activities carrying smart phones at different positions. Based on the analysis of this data set, we show that these sensors, except the magnetometer, are each capable of taking the lead roles individually, depending on the type of activity being recognized, the body position, the used data features and the classification method employed (personalized or generalized). We also show that their combination only improves the overall recognition performance when their individual performances are not very high, so that there is room for performance improvement. We have made our data set and our data collection application publicly available, thereby making our experiments reproducible.

## 1. Introduction

Physical activity recognition using wearable sensors has enabled the scientific community to develop novel applications, especially in the area of healthcare and assisted living [1,2]. In recent years, smartphones have been used for activity recognition, because they are readily equipped with several sensors useful for activity recognition, such as motion and location sensors. Moreover, they are carried by almost everyone in their daily lives.

So far, among these smartphone sensors, the accelerometer has received the most attention in the activity recognition research. However, in recent years, other sensors, like the gyroscope and magnetometer, have been combined with an accelerometer with the aim of improving activity recognition performance [3,4]. However, to the best of our knowledge, there is no study investigating the performance of these sensors in detail considering different feature sets, classifiers, different phone carrying positions, both individually, as well as in combination. Moreover, using an additional sensor causes more energy consumption, which can be problematic for an energy-limited device, like a smartphone [5]. Hence, such a detailed analysis can help in deciding when to best combine these motion sensors. Therefore, there is a need to study the role of these sensors in detail. In particular, we focus on this research question: “How and when are various motion sensors, which are available on a smartphone, best used for better recognition performance, either individually or in combination?”

Some researchers have already investigated the combination of various motion sensors in activity recognition [3,4]. For example, in [3], the authors use a gyroscope in combination with an accelerometer and report an increase in the recognition performance (accuracy) by 3.1%–13.4% for some activities. On the other hand, in [4], the authors claim that the addition of a gyroscope to an accelerometer does not add any value to the recognition performance (accuracy). These two papers probably report different results, due to their different experimental setups.

However, these previous studies have explored the combination of various motion sensor only in some specific scenarios. In order to answer our question, we study the role of these sensors in detail in different scenarios. We defined three different evaluation scenarios in order to cover the most commonly used scenarios in the previous studies. These scenarios are:

1. the position-aware evaluation scenario, where these sensors are evaluated on a single position;
2. the position-unaware evaluation scenario, where these sensors are evaluated on multiple positions;
3. the personalized evaluation scenario, where the classification methods are trained and tested for a specific user with his or her own data.

Moreover, we evaluate the recognition performance with four different sensors: an accelerometer, a gyroscope, a linear acceleration and a magnetometer. The linear acceleration sensor is a virtual sensor, derived from the accelerometer by removing the gravity component [6]. These sensors are selected because they were used in previous activity recognition studies [3,4,6,7]. The main focus, however, is on the accelerometer and the gyroscope, since these are the mostly-used sensors in similar studies.

In particular, the goal of this paper is to provide a detailed analysis of whether to fuse data from multiple sensors. We believe that our effort will assist the readership and this will save time for future studies by not repeating the same experiments. This study can be used as a basis for making design decisions about when and how to combine these sensors for better activity recognition. The main contributions and highlights of this paper are as follows:

- To the best of our knowledge, we are the first to do such an extensive analysis of the role of these sensors in activity recognition, both when they are used alone, as well as in combination with each other. We evaluate them for five body positions using nine classifiers. Moreover, we have used four feature sets in our evaluations, which are all used in the state-of-the-art. They have low or medium complexity and are suitable for running on smartphones [8]. We recognize seven physical activities, commonly used in the state-of-the-art.
- We also investigate the recognition performance when the training and testing data is coming from a single user (personalized classification) or from multiple users (generalized classification). Moreover, we use both position-aware and position unaware classification for our evaluation scenarios.
- We make our data set and our data collection application publicly available for future research in this domain [9].

The rest of the paper is organized as follows. We describe related work in Section 2 and the data collection process in Section 3. The data preprocessing is described in Section 4 and our evaluation approach in Section 5. We discuss the performance evaluation in Section 6. Finally, we describe our conclusions and future work in Section 7.

## 2. Related Work

While the use of a smartphone accelerometer in activity recognition has been extensively studied [10], the use of a gyroscope and a magnetometer in activity recognition is yet to be explored in detail. Some researchers have evaluated the effect of amending accelerometer-based activity recognition with gyroscope data [3,4]. However, the gyroscope alone has not received much attention for physical activity recognition, especially in terms of comparing its performance with an accelerometer, a magnetometer and combinations of all these sensors.

Studies where a gyroscope was either used alone or in combination with an accelerometer exist for many applications related to activity recognition. For example, for gait analysis [11], fall detection [12] and gesture recognition [13,14], gyroscopes have been used, either alone or in combination with an accelerometer. Moreover, an accelerometer and a gyroscope were combined to detect certain dangerous driving behaviors in [15,16]. In [17], a gyroscope and an accelerometer were combined for estimating the intensity of physical activity. In [18], the combination of an accelerometer and a gyroscope was used to recognize different tai chi movements. In [19], the combination is used for the motion monitoring of patients with disorders, such as Parkinsons Disease, epilepsy and stroke [14].

For activity recognition, there is earlier work in which motion sensors (accelerometer, gyroscope, magnetometer and linear acceleration) are used either alone or in different combinations. However, the merit of the individual sensors is often not evaluated in detail. In [20], the combination is used for fall detection and activity recognition using an SVM classifier [21]. In [22], the combination is used to detect step count and also to recognize different modes of motion. In [23], the combination is used for detecting three transportation modes, such as walking, riding a car and a train, and improvement in accuracies is reported. In [24], the combination is used to detect postures (laying, sitting and standing), locomotion (walking) and transitions (sit-to-stand, stand-to-sit). In [25], the combination is used to recognize different physical activities using three selected classifiers. In [7], the authors compare different classification methods for recognizing physical activities using data from three sensors (accelerometer, gyroscope and magnetometer). In [26], the authors combine multiple accelerometers and gyroscopes (on a sensor board) to recognize different activities and to evaluate the displacement effect on these sensors. The work in [27,28] shows the potential of a magnetometer sensor in activity recognition when used alone. A linear acceleration sensor is used in [6] for recognizing activities.

There are few studies [3,4,29,30] that are similar to our study. For example, the authors in [4] used a combination of an accelerometer and a gyroscope and claimed that the gyroscope adds no value to the overall recognition performance. They used naive Bayes, decision tree (C.45) and k-nearest neighbor (KNN) for classification and collected data on multiple body positions. The authors in [3] also used a combination of an accelerometer and a gyroscope and reported a 3.1%–13.4% increase in recognition accuracy for some of the evaluated activities. However, they did these experiments only with a KNN classifier. They considered only the pocket position, except for the jogging activity, for which an arm position was used. In [29], the authors used an accelerometer, a magnetometer, a gyroscope, linear acceleration and gravity in combination. Though this combination performed slightly better than the accelerometer alone, the paper does not discuss the role of the individual sensors. Therefore, it is not clear which sensors contributed (and how much) to the improvement in the activity recognition. The authors in [30] evaluated the gyroscope, the accelerometer and magnetometer using a single classifier (principle component analysis). They used a sensor board, equipped with these sensors. Moreover, they have only one participant data set for the arm position. They show that the accelerometer and gyroscope can recognize walking and jogging activities, whereas the magnetometer performs poorer, without reporting any accuracy results. We further investigate the role of smartphone sensors in detail using those studies as a starting point.

Existing work uses the accelerometer as the lead sensor, while attributing only a supporting role to the gyroscope [3,4,29]. In contrast, we evaluate these sensors individually, as well as in combination with each other, thereby identifying their individual contributions in the activity recognition. We performed an initial study [31], where we evaluated three motion sensors (the accelerometer, the gyroscope and magnetometer) and showed that they have the potential to take lead roles in the activity recognition process when used alone. However, we evaluated these sensors in only one scenario. In our current study, we consider multiple evaluation scenarios, as discussed in Section 6. This enables us to demonstrate where and when a sensor performs better than the others and when they perform better in combination. Moreover, we evaluate the sensors using nine classifiers. This evaluation is done on five different body positions with ten participants, where in the previous study, the number of participants was limited to four. This enables us to make more confident claims about our reported results. We also compare the results of the linear acceleration sensor with the accelerometer and the gyroscope. Additionally, we show how to improve the performance of a magnetometer in the activity recognition process, if the correct feature set is used.

## 3. Data Collection

**Figure 1.**
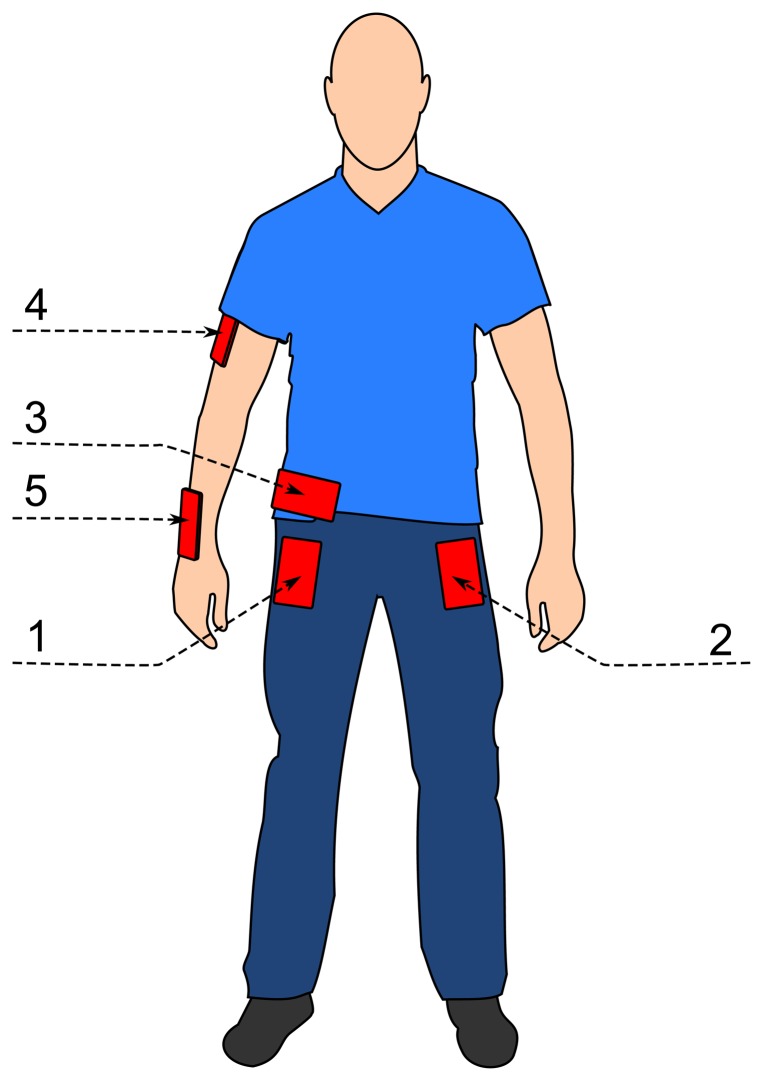
Overview of the phone positions on a participant.

In the data collection experiments, we collected data for seven physical activities. These are walking, running, sitting, standing, jogging, biking, walking upstairs and walking downstairs, which are mainly used in the related studies, and they are the basic motion activities in daily life. There were ten participants involved in our data collection experiment, who performed each of these activities for 3–4 min. All ten participants were male, between the ages of 25 and 30. The experiments were carried out indoors in one of the university buildings, except biking. For walking and running, the department corridor was used. For walking upstairs and downstairs, a 5-floor building with stairs was used. Each of these participants was equipped with five smartphones on five body positions (as shown in Figure 1):

1. one in their right jeans pocket;
2. one in their left jeans pocket;
3. one on the belt position towards the right leg using a belt clip;
4. one on the right upper arm;
5. one on the right wrist.

The first three positions are commonly used by people carrying smartphones. The fourth position is usually used when activities like jogging are performed. However, we used this position for all activities to see its role on the performance. A smart-watch was simulated with the fifth position, as smart-watches are coming into the market these days [32]. It is important to note that the positions for these smartphones on participants' bodies are fixed. For these experiments, we used a Samsung Galaxy SII (i9100) smartphones [33].

The orientation of the smartphones was portrait for the upper arm, wrist and two pockets and landscape for the belt position. The data was recorded for all five positions at the same time for each activity, and it was collected at a rate of 50 samples per second. This sampling rate (50 samples per second) is enough to recognize human physical activities, as we show in our previous study [31]. Moreover, in the state-of-the-art, frequencies lower than 50 samples per second have been shown to be sufficient for activity recognition [3,4].

For data collection, we adapted our own data collection app from our previous study [31] by adding the linear acceleration sensor. The data was collected for an accelerometer, a gyroscope, a magnetometer and a linear acceleration sensor.

## 4. Preprocessing Data

We divided the collected data into small segments for feature extraction using the sliding window approach. The selection of an appropriate window size is important, and different values can be set for it. However, we selected a sliding window of two seconds based on previous studies [3,34], since it was shown that a window size of two seconds was an effective and sufficient value for a reasonable activity recognition performance. We used the sliding window approach with an overlap of 50% (1s overlap here), based on the reporting of previous studies [3,34,35]. Even though different overlap values can be used, an overlap of 50% has been shown to produce reasonable results [3,34,35]. Based on our two-second window, we have 180 window segments for each activity for a single user at a single position. For all ten participants, we have 1800 segments for each activity at a single position. In some scenarios, we combined data from three positions, in which case, we have 5400 segments for each activity over all the three positions.

Each of these sensors has three dimensions, that is the x-axis, y-axis and z-axis, and their respective values are reported along these axes. Most of the existing work assumes a fixed orientation while evaluating different classification algorithms [3]. However, the recognition performance of these sensors may be affected by the orientation changes if the classification algorithms were trained only for a specific orientation [36], leading to a drop in performance. In order to minimize such effects caused by orientation changes, we added a fourth dimension to the existing three dimensions of each sensor, called the magnitude of a sensor. This choice was motivated by the work done in [36] about orientation-independence in activity recognition, because the magnitude feature is less sensitive to orientation changes. The magnitude for each sensor is calculated using the following formula:

(1) $$\text{magnitude}=\sqrt{x^{2}+y^{2}+z^{2}}$$

Based on this addition, we had four dimensions for each sensor *i.e.*, (*x*, *y*, *z*, *magnitude*). For each sliding window with a 50% overlap, we extracted different features, as listed in Table 1. We used four feature sets based on the fact that all of them have low or medium complexity and are suitable for running on smartphones [8]. Three of these feature sets are comprised of time domain features, and one set is comprised of frequency domain features.

The main reason for selecting these four feature sets was to validate our results using different types of features for more confident and generic results. Though time domain features are computationally cheap compared to frequency domain features, due to the expensive Fourier transformation [4,8], we still chose one feature set from the frequency domain to validate our results in a generic way For example, feature sets FS1, FS2 and FD3 include time domain features, and feature set FS4 includes frequency domain features. The grouping in Table 1 is motivated by the fact that these features are used in similar ways in the previous studies, as reported in [8,34]. For example, FS1 is used in [8,31,34]. Moreover, the combination of zero crossings and the root mean square is used for gesture recognition, as reported in [8]. We selected these two features, because they are less sensitive to orientation changes (in the case of the accelerometer and the gyroscope) and to direction changes (in the case of a magnetometer). However, our initial evaluations with this combination did not show encouraging results in recognizing our seven activities. That is why we added the median feature to FS2, which has been shown to produce reasonable results for activity recognition [8]. Moreover, variance was added to FS3 to deal with direction changes in a magnetometer. We combine the two frequency domain features in FS4 for better activity recognition results. All these features were selected, because they have very low or medium computational and storage complexity. The low complexity makes these features suitable for running on smartphones, as shown in Table 2 [8]. For a detailed definition of the complexity levels used in this table, such as ‘low’, ‘very low’ and ‘moderate’, readers can refer to [8], where these are explained in detail.

All of the these features are extracted over a sliding window of 2 seconds with a 50% overlap. All of these features have been used in different studies, as reported in [8]. They are defined as follows:

- Zero crossings: this is defined as the number of points where a signal crosses through a specific value corresponding to half of the signal range [8]. In our case, that specific point is the mean of a window segment.
- Root mean square value: The root mean square (RMS) of a signal, *x*, that represents a sequence of *n* discrete values *x*_1_, *x*_2_, …, *x_n_* is obtained using the following formula and can be associated with meaningful context information [8]: $RMS=\sqrt{\frac{x_{1}^{2}+x_{2}^{2}+\ldots x_{n}^{2}}{n}}$.
- Spectral energy: The energy of the signal can be computed as the squared sum of its spectral coefficients normalized by the length of the sample window [8].
- Mean: This is the average value of all sample values in the sample window [8].
- Variance: This is the average of the squared differences of the sample values in a sample window from its mean [8].
- Standard deviation: This is the square root of variance [8].
- Median: This is defined as the value that divides the higher half of the sample window from the lower half [8].
- Sum of FFT coefficients: This is defined as the sum of the number of FFT coefficients [8]. In our case, we take the first five FFT coefficients, as these contain the main frequency components.

We started from the simplest possible situation. That is why we did not normalize our features. We defer the evaluation of the effect of normalization to future work. Furthermore, we did not explicitly calibrate our sensors. We briefly evaluated the calibration of the accelerometer, and we found that it was factory-calibrated with a reasonable accuracy.

## 5. Evaluation Approach

In order to analyze the preprocessed data, we used the WEKA machine learning tool (Waikato Environment for Knowledge Analysis) [37,38]. There are different classification and preprocessing algorithms available in this tool. These algorithms use the data in the ARFF (attribute-relation file format) file as the input. Therefore, the preprocessed data (extracted features) were converted to ARFF file format, which is a WEKA file format. This is an ASCII text file format, and it defines a data set in terms of a relation (table) made up of attributes (columns of data) [37]. Information about the structure of the relation is stored in the ARFF header, while the actual data is represented in the body of the ARFF file as lines with comma-separated attribute values.

Then, we applied different classifiers on these data to evaluate their performance. We used the 10-fold stratified cross-validation technique to evaluate different classifiers. In stratified cross-validation, each fold or part of data contains all classes in equal proportion to ensure fairness [38]. In 10-fold cross-validation, the data set is divided into 10 bins. Out of these ten bins, nine (90%) are used for training and one (10%) for testing. This process is repeated ten times, each time with a different bin for testing, thereby using all data, both for training and testing. We selected nine classifiers from different types of classification algorithms, as listed in Table 3. We use the short notations in Table 3 for these classifiers in the next sections. We selected these classifiers, as they have been used in the state-of-the-art for activity recognition [3,10,39]. Some of these classifiers have been implemented on smartphones for activity recognition. For example, KNN and naive Bayes are used in [40] for online activity recognition on smartphones. The decision tree classifiers are implemented on smartphones in [4,41]. The SVM classifier is implemented on smartphones in [20,42]. The NNGEand PARTclassifiers are notable exceptions, which are not used mainly for physical activity recognition. NNGE and PART are rule-based classifiers, which create certain decision rules in the training phase, and then, test data is classified based on these rules. This behavior makes these classifiers faster for the testing phase, and we believe they will be suitable for running on smartphones, especially where the training is a onetime task and testing is a continuous process after that. NNGE is a rule-based classifier based on K-nearest neighbor, whereas PART is based on decision trees. The details on the implementation of these classifiers can be found in WEKA documentation [38].

We used all these classifiers in their default settings in WEKA 3.7.10. We did not perform any optimizations, because we are more interested in the relative roles of our three sensors in the classification process. This also means that our reported absolute accuracies may not be the best possible and may be improved further. We omitted optimization to make these experiments easily reproducible.

## 6. Performance Analysis and Discussion

In this section, we discuss the role of the smartphone sensors in terms of recognition performance. We use accuracy, also known as the true positive rate (TP rate or TPR), as our performance metric. The TPR of a classifier is the proportion of correctly classified examples (window segments) of a specific class out of all its examples [43]. In Section 6.1, we discuss the role of an accelerometer and a gyroscope in different situations. In comparing these sensors, we consider a performance difference of anything less than 2% as equal, and any difference beyond this margin is considered relatively better. This is only done to present the results in a simpler way and to ignore insignificant performance differences. This rule is followed for presenting the results in all of the following sections.

The role of the linear acceleration sensor and the magnetometer is discussed in Section 6.2 and 6.3, respectively. In Section 6.4, we discuss the lessons learned based on all of our evaluations and the limitations of this study.

### 6.1. The Role of an Accelerometer and a Gyroscope

In order to understand the performance of an accelerometer, a gyroscope and their combination, we evaluated them using nine classification methods on five different body positions. We performed these evaluations for them individually, as well as for their combination. In order to cover different evaluation scenarios, as used in the previous studies [3,4,44], and to understand their results, we create three evaluation scenarios as shown in Table 4. For example, the authors in [3] use data from a single position (jeans pocket) for their evaluation. In Scenario A, we use data from a single position for training and testing classifiers. Three feature sets (FS1, FS2, FS4) are used for evaluation in this scenario. The authors in [4] combine data from multiple positions (pants pocket, shirt pocket, hand, hang bag) for training and testing the classification methods. In Scenario B, we combine data from multiple positions (upper arm, wrist and pockets) to train and test different classification methods. We used FS1, FS2 and FS4 for this scenario. In [44], the authors use data from a single user for training and testing a classifier, making it a personalized classifier. Therefore, in Scenario C, we train and test our selected classifiers with data from a specific single user, making it personalized. With these three scenarios, we cover the commonly used situations in which an accelerometer and a gyroscope are used for activity recognition in the literature study.

#### Scenario A

We evaluated the accelerometer and the gyroscope for seven physical activities using nine classifiers and three feature sets. This was performed for all five positions individually. Next, we discuss these evaluation results for each of the seven activities. We discuss the activities in the following order: walking downstairs, walking upstairs, walking, jogging, biking, sitting and standing.

**Figure 2.**
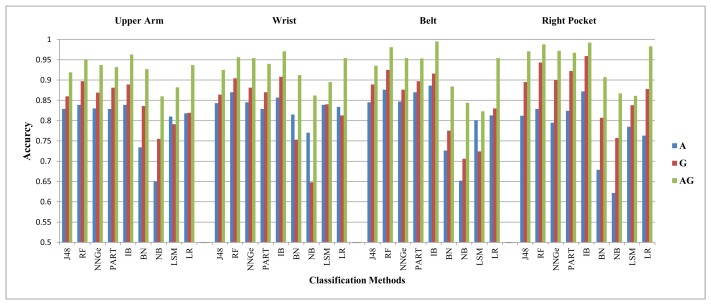
Recognition performance for the walking downstairs activity using FS2.

**Figure 3.**
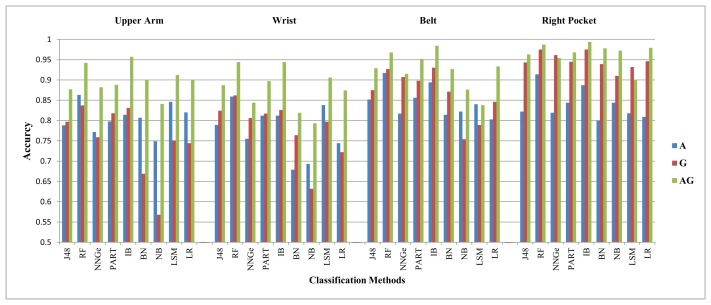
Recognition performance for the walking upstairs activity using FS2.

We summarize the evaluation results for both the walking downstairs and walking upstairs activities in Tables 5 and 6. These tables show which sensor is in the leading role for recognizing a specific activity at a specific position, while using a specific classifier and feature set. In these tables, A stands for an accelerometer and G stands for a gyroscope. Each cell in this table shows which sensor performed better for which feature sets for a specific classification method at a specific body position. For example, the first cell in Tables 5 is *G(FS2)*/**A**(**FS4**)/E(FS1). This means that for the J48 classifier on the upper arm position, the accelerometer performed better than the gyroscope using FS4, whereas the gyroscope performed better than the accelerometer using FS2. The E means equal within the 2% difference margin, and it shows that both these sensors performed equally on FS1. This format is followed in Tables 7 and 8. In Table 8, LA stands for the linear acceleration sensor. For clarification purposes, we highlighted the G symbol as italic and A as regular bold. We did not include the LSMresults in these tables for feature set FS4, because the resulting performance is unusably low. Moreover, it is important to note that the performance trends were similar for the right and left pocket positions, so we just show the right jeans pocket in all of the next tables and graphs for simplicity. The performance for walking downstairs and walking upstairs activities is also shown in Figures 2 and 3.

Though it is hard to make one generic statement based on these results, they do show some trends:

- For the walking downstairs activity, the gyroscope performs better than the accelerometer in most cases at all positions, especially using FS2 and FS4. The gyroscope performs better by different margins at different positions using different feature sets. For example, at the right pocket position, on average, the gyroscope performs better than the accelerometer by 10.2% using FS2. This average is 7.2% for FS1 and 4.6% for FS4. We only take the average for those classifiers where a sensor performs at least 2% better than the other sensor. These margins for the gyroscope at the belt position are 4% for FS2 and 8% for FS4. For FS1, the accelerometer performs better than the gyroscope by an average of 12.4% at the belt position. In a few situations, they perform equally. For the walking downstairs activity, the detailed analysis on the performance differences of these sensors at different positions with different classifiers and feature sets is given in Table A.1 in the Appendix.
- For the walking upstairs activity, the gyroscope performs better than the accelerometer in most cases at all positions, except at the upper arm position, especially using FS2 and FS4. The gyroscope performs better by different margins at different positions using different feature sets. For example, at the right pocket position, on average, the gyroscope performs better than the accelerometer by 10.8% using FS2. These averages at the right pocket are 4% and 3.8% using FS1 and FS4, respectively. At the belt position, these averages for the gyroscope are 4.9% for FS2 and 7.6% for FS4. For FS1, the accelerometer performs better than the gyroscope by an average of 11.9% at the belt position. In a few situations, they perform equally. For the walking upstairs activity, the detailed analysis on the performance differences of these sensors at different positions with different classifiers and feature sets is given in Table A.2 in the Appendix.
- In terms of different classifiers, the gyroscope performs better than the accelerometer in most cases with decision tree-based classifiers (RF, PART, J48) or k-nearest neighbor-based classifiers (IB, NNGE), especially at the pocket and belt positions using FS2 and FS4.
- In terms of body positions, the gyroscope mostly takes the leading role in recognizing walking downstairs activity at the upper arm using FS1 and FS2, at the right pocket using FS1 and FS2, at the belt using FS2 and FS4 and at the wrist using FS2 and FS4. On the other hand, the accelerometer mostly takes the leading role at the upper arm using FS4 and at the belt position using FS1. For recognizing the walking upstairs activity, the gyroscope mostly takes the leading role at the right pocket using FS1 and FS2 and at the belt using FS2 and FS4, whereas the accelerometer takes the reading role at the upper arm using FS2 and FS4 and at the belt using FS1.
- The results show that the performance of the accelerometer and the gyroscope for recognizing the walking upstairs and walking downstairs activities depend on the body positions, the data features and the classification methods being used. Overall, the gyroscope performs better than the accelerometer in most of the situations.
- Moreover, in almost all of these cases, the combination of these sensors perform better than the maximum of their individual performances in recognizing the walking upstairs and walking downstairs activities. For the walking downstairs activity, the average improvement at all positions for all classification methods is 13.2%, 7.5% and 10% using FS1, FS2 and FS4, respectively. For recognizing the walking upstairs activity, these values are 9.1%, 6% and 7.2% using FS1, FS2 and FS4, respectively. The performance differences for these two activities at different positions with different classification methods and feature sets are shown in detail in Table A.5 and Table A.6 in the Appendix.

The margin between the gyroscope and the accelerometer performance varies in different situations. This performance variation is not clear from Tables 5 and 6. Therefore, in order to show a concrete example of the performance differences between these two sensors and their absolute TPR values, we present two example graphs for FS2. The performance for the walking downstairs and walking upstairs activities is shown in Figures 2 and 3, respectively. In these graphs, A stands for accelerometer, G for gyroscope and AG stands for their combination. The performance graphs in the rest of the paper follow the same order and labeling, unless otherwise specified. It is clear from these graphs that these two sensors take lead roles with different margins depending on the body position and classification method. The improvement of their combination is also visible in these two graphs. The amount of improvement is also different depending on the body position and classification method used. However, the trend of improvement is consistent for all positions and classification methods. The exact values for these performance differences are given in Tables A.1, A.2, A.5 and A.6 in the Appendix.

**Figure A.1.**
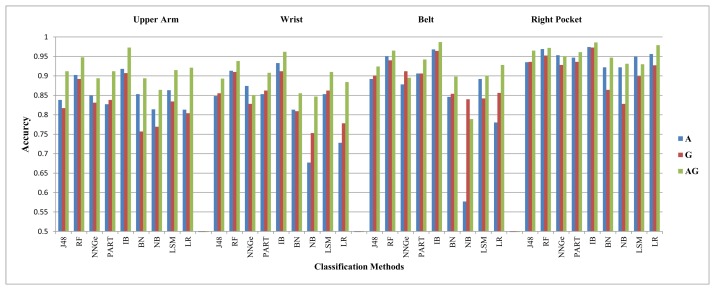
Recognition performance for the walking activity at all four positions using FS2.

For the walking activity, it is mainly the accelerometer that takes the leading role. For example, it performs better than the gyroscope at all positions by an average of 6.3% and 3.7% using FS1 and FS2, respectively. However, the gyroscope also performs reasonably well in recognizing the walking activity, and in some cases, it even takes the leading role. For example, it takes the lead role at the belt position using FS4 and performs better than the accelerometer by an average of 14.2% for all classifiers. Moreover, in some cases, they perform equally, as shown in Table 7. Their relative ranking in terms of leading role is shown in Table 7. The percentage difference between these two sensors for the walking activity with different classification methods and feature sets is given in Table A.3 in the Appendix. Moreover, in order to show the difference between their performances, we present one graph as an example, as shown in Figure A.1 in the Appendix. It shows the classification performance for the walking activity on all positions using feature set FS2.

In most cases, the combination of the two sensors did not bring significant improvement to the overall performance. This improvement ranged from 2% to 24% in different situations, but the overall average for all classification methods at all positions was 3.7%, 3.3% and 3.8% using FS1, FS2 and FS4, respectively. As we pointed out earlier, that when one of the sensors performs with higher accuracy, then there is less room for improving that accuracy by combining another sensor to it. In this case, both the sensors performed with higher accuracy individually. In situations where they performed with low accuracies, their combination improved the overall performance. For the walking activity with different classification methods and feature sets, the performance differences of the combination of these sensors are shown in Table A.7.

**Figure A.2.**
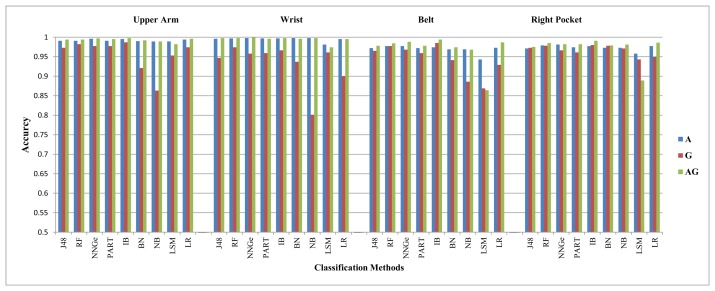
Recognition performance for the jogging activity on all four positions using FS2.

For the jogging and biking activities, the accelerometer performs almost always slightly better than the gyroscope irrespective of the body position, the feature set and the classification method. However, the gyroscope also performs reasonably well for these two activities. The performance graphs for the jogging and biking activities using FS2 are shown in Figures A.2 and A.3, respectively, in the Appendix. The same trends were observed for the other feature sets, too. We calculated the average performance for all classification methods at all positions for these two activities. For the jogging activity, the accelerometer performed better than the gyroscope by an average of 5%, 3.2% and 4.9% using FS1, FS2 and FS4, respectively. For the biking activity, these averages are 6.4%, 5% and 6.7% using FS1, FS2 and FS4, respectively. The combination of a gyroscope and an accelerometer follows the same rule for bringing improvement in the overall performance, such that if there is room for improvement, it brings more significant improvement. In this case, both of these sensors performed with higher accuracy, so there is no significant improvement in the overall accuracy when they are combined.

**Figure A.4.**
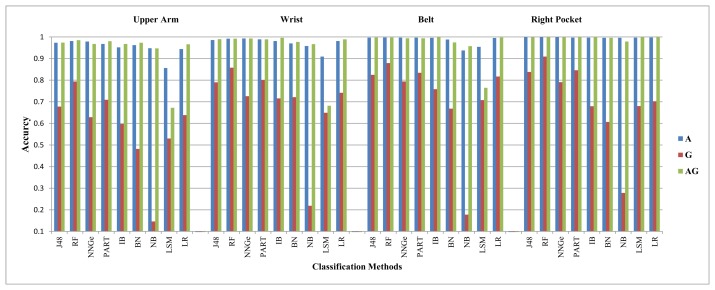
Recognition performance for the standing activity at all four positions using FS2.

The accelerometer always performs better than the gyroscope for recognizing the sitting and standing activities irrespective of the body position, the classification method and the feature set. Even though the difference between their performances varies in different situations, the accelerometer always takes a lead role here. For the standing activity, the accelerometer performs better than the gyroscope at all positions by an average of 41%, 30.4% and 32.8% using FS1, FS2 and FS4, respectively. For the sitting activity, these averages are 14.7%, 20.4% and 17.3% using FS1, FS2 and FS4, respectively. The performance of these two sensors and their combination for FS2 is shown Figures A.4 and A.5, respectively, in the Appendix. The reason that the gyroscope has a low performance is that it is unable to differentiate between the sitting and standing activity. The same was shown in our previous study [31]. The low performance for the gyroscope could be possible due to the lack of gravity. The gravity component makes it easier for the accelerometer to differentiate between the sitting and standing activities. We observe a performance drop for these two activities when we removed the gravity component from the accelerometer to get the linear acceleration values, as discussed in Section 6.2, which shows that the gravity plays an important role in differentiating these two activities. The combination of the two sensors does not bring significant improvement here, because the accelerometer recognizes these two activities with high accuracy, thereby leaving less room for any further improvement. However, from the gyroscope perspective, its combination with an accelerometer does bring significant improvement to the overall recognition performance and covers its weakness of confusing these two activities.

**Figure A.6.**
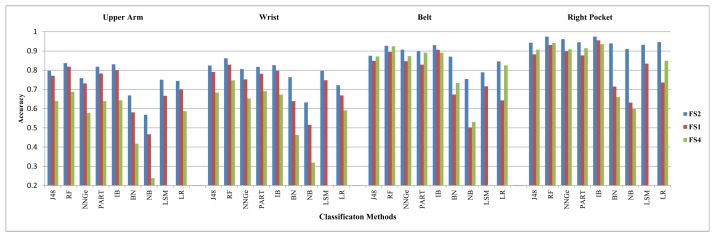
Gyroscope TPR for the walking upstairs activity for all four positions and three feature sets.

In order to see the relative performance of different sensors at different positions, we plot the performance of these sensors on four body positions using three feature sets. Figures A.6 and A.7 in the Appendix show the relative performance of the gyroscope and the accelerometer across different positions, respectively. These graphs only show the classification accuracies for the walking upstairs activity. It is clear from these graphs that the change in performance for the gyroscope on different body positions is more visible than that of the accelerometer. The gyroscope performance is better at the pocket positions compared to other positions and usually lower at the upper arm and wrist positions. In general, the performance of the gyroscope gets better as we successively move from the upper arm to the wrist to the belt to the pocket positions. However, for the biking and jogging activities, the gyroscope performance remained almost the same at all positions. For the accelerometer, this change was lower compared to the gyroscope. Moreover, we observe this change only for the walking, walking upstairs and walking downstairs activities. For the rest of the activities, its performance remained almost the same at all positions. In general, it performed well at the pocket positions, like the gyroscope.

In terms of feature sets, there are different trends. In general, both of the sensors performed better with feature sets FS1 and FS2 compared to FS4. As far as FS1 and FS2 are concerned, the gyroscope performed better in some situations with feature set FS2 compared to FS1, especially for recognizing the walking downstairs and upstairs activities at all positions. For other activities, there were mixed results. On the other hand, the accelerometer performed almost the same for both feature FS1 and FS2 with a few exceptions. Overall, the performance of the accelerometer was more consistent compared to that of the gyroscope with changing body positions and feature sets.

In order to see the effect of the extra magnitude dimension for these two sensors (as explained in Section 4), we modified this scenario by removing the fourth magnitude dimension from the features. After that, we evaluated these two sensors with the original three dimensions on all five positions using FS1 and FS2. However, we observed almost the same trends, although absolute values of the recognition performance varied in different situations. We do not show their performance graphs in this scenario, because of no significant changes in the comparative analysis of the two sensors. Moreover, we compared the results from both cases, such that with and without extra magnitude dimension, they were, in most cases, almost the same. In a few cases, the classification accuracies for sensors with extra magnitude were slightly higher or lower than that of without extra magnitude. However, the average difference for all classification methods at all positions was less than 1% in almost all situations for FS1 and FS2, except for the walking downstairs and the walking upstairs activities. For these two activities, on average, the extra magnitude performed better by a 3%–4% margin.

#### Scenario B

First, we combined data from three positions, upper arm, wrist and right pocket, for training and testing classifiers, and then, we added the left pocket to it. We did not select the belt position, because it has a different orientation than the other positions, and we wanted to keep the orientation fixed. First, we selected three different types of positions, so that is why we did not include the left pocket.

**Figure 4.**
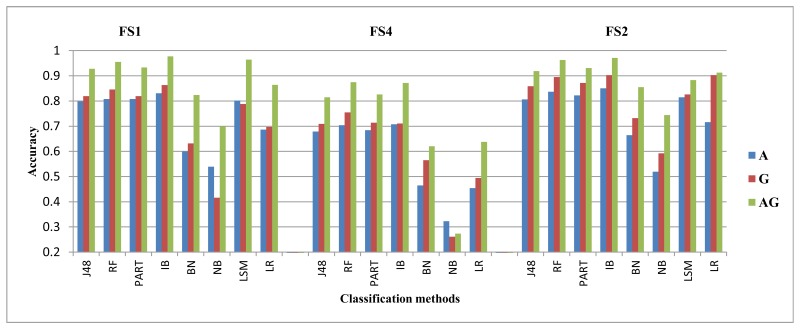
The recognition performance for the walking downstairs activity in Scenario B.

**Figure 5.**
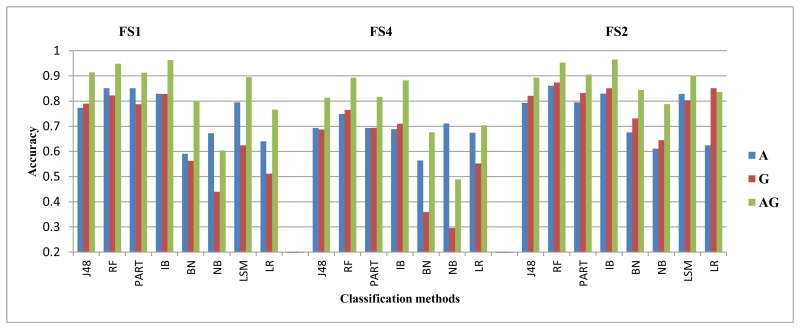
The recognition performance for the walking upstairs activity in Scenario B.

We evaluate the combination of the three positions first. For this combination, the accelerometer performs better than the gyroscope by high margins for all activities, except the walking, walking downstairs and walking upstairs. The average of these performance margins is within the range of 6%–10%, 10%–17%, 15%–21% and 33%–47% for the jogging, biking, sitting and standing activities, respectively. For the walking activity, these two sensors perform slightly better than each other in different situations, within an average performance difference of 1%–4%. The gyroscope performs slightly better than the accelerometer for the walking downstairs activity and, in some case,s for the walking upstairs activity, as shown in Figures 4 and 5. For example, using FS2, the gyroscope performs better than the accelerometer by an average value of 6.9% for recognizing the walking downstairs activity and by an average value of 4.9% for recognizing the walking upstairs activity. The gyroscope performs very poorly with the sitting and standing activities, like in Scenario A. For the walking, walking upstairs and walking downstairs activities, the performance differences of these sensors with different classification methods and feature sets are shown in Table A.4 in the Appendix.

Their combination did not improve the overall performance for all activities, but walking, walking upstairs and walking downstairs. For these three activities, the combination of these sensors brings significant improvement. For example, using FS1, their combination improved the overall recognition performance for the walking, walking upstairs and walking downstairs activities by an average value of 5.1%, 9.8% and 14.1%, respectively. This result partially confirms the results in [4]. In [4], the authors combine data from four different body positions and report that the addition of a gyroscope to the accelerometer yields no performance increase. However, that statement does not hold for the walking, walking upstairs and walking downstairs activities, since we observe improvement for these activities. These different conclusions can result from the fact that we use different body positions than the ones used in [4]. For the walking, walking upstairs and walking downstairs activities, the performance differences of the combination of these sensors with different classification methods and feature sets are shown in Table A.8 in the Appendix. Moreover, we evaluated the data combination of the four positions, such as the upper arm, the wrist, the left and the right pocket. Though the absolute value of performance differences were slightly different in this case, we observed similar trends in comparing these sensors for activity recognition as in the combination of three positions.

As we did for Scenario A, we modified this scenario by removing the extra magnitude dimension from the two sensors and evaluated them for FS1 and FS2. We observe almost the same trends without this magnitude dimension as we observe with the extra magnitude dimension.

#### Scenario C

**Figure A.8.**
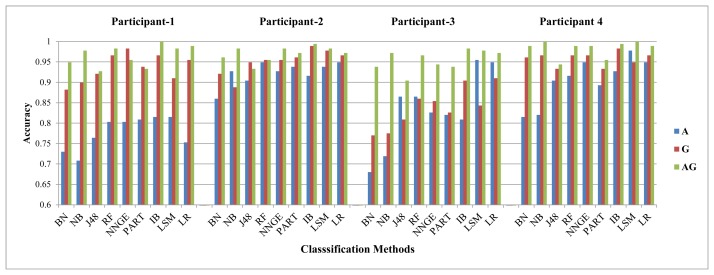
Recognition performance for the walking downstairs activity in Scenario C.

We evaluated the gyroscope and the accelerometer for individual participants only at the right pocket position while using personalized classification using FS1. We observe similar trends in this scenario as Scenario A, in terms of comparing the gyroscope and the accelerometer. The gyroscope performed slightly better than the accelerometer for the walking upstairs and walking downstairs activities. For walking, jogging and biking activities, they performed almost the same. For sitting and standing activities, the accelerometer performed better than the gyroscope. The difference between accelerometer and gyroscope TPR varied between different participants. This variation was visible for the sitting, standing, walking upstairs and downstairs activities and negligible for all other activities. For the walking downstairs activity, the gyroscope performed better than the accelerometer with an average of 7.2% ranging from zero to 17% for different participants. For the walking upstairs activity, this average was 3.4% ranging from zero to 11% for different participants. On the other hand, for the sitting activity, the accelerometer performed better than the gyroscope by an average of 25% ranging from 5% to 36% for different participants. For the standing activity, this average was 10%, ranging from 2% to 24% for different participants. In most situations, the gyroscope and the accelerometer performed relatively better than their performances in Scenarios A and B, because of the personalized data set. Due to this effect, their combination did not improve the overall performance, except with slight improvements for the walking upstairs and walking downstairs activities. The combination of these two sensors improved the overall recognition performance by an average value of 2% and 2.7% for the walking upstairs and the walking downstairs activities, respectively. These values are very low compared to those in the Scenario A and B. Though we evaluated all participants, for simplicity, we show the results only for four participants, as an example. The results are for recognizing the walking downstairs and walking upstairs activities are shown in Figures A.8 and A.9, respectively, in the Appendix.

### 6.2. The Role of a Linear Acceleration Sensor

In this section, we discuss the role of the linear acceleration sensor. This sensor has been recently used in activity recognition, because it is less sensitive to the orientation effects [6]. We wanted to see how it behaves in comparison with the accelerometer and the gyroscope. For this purpose, we evaluated this sensor in the following two scenarios, as shown in Table 9.

#### Scenario D

We observe performance trends similar to the accelerometer in Scenario A, with a few exceptions. It takes the leading role where the accelerometer was in the leading role compared to the gyroscope. Table 8 shows its position in terms of performance in comparison with the accelerometer at different body positions using different feature sets. This table shows the generic trends of all classifiers based on their average performance difference for these sensors for all seven activities at four body positions. The linear acceleration sensor performs better than the accelerometer for the walking, walking upstairs and walking downstairs activities at the upper arm position. In all other situations, mostly the accelerometer performs better or they have equal performance.

#### Scenario E

We found that the linear acceleration sensor performs almost the same or poorer than the accelerometer for all of the activities. The accelerometer performed better than the linear acceleration by an average value of 28%, 12%, 8%, 6% and 5% for the standing, sitting, biking, walking upstairs and walking downstairs activities, respectively. The average was taken for all classification methods using FS1, FS2 and FS4. The possible cause for the linear acceleration sensor to perform poorly with the sitting and standing activities could be the lack of a gravity component, which plays an important role in differentiating the two stationary postures of sitting and standing for the accelerometer. For the walking and jogging activities, on average, these two sensor performed almost equally. As far as its comparison with the gyroscope is concerned, the trends remained very similar to those for the accelerometer in Scenario B.

### 6.3. The Role of a Magnetometer

**Figure 6.**
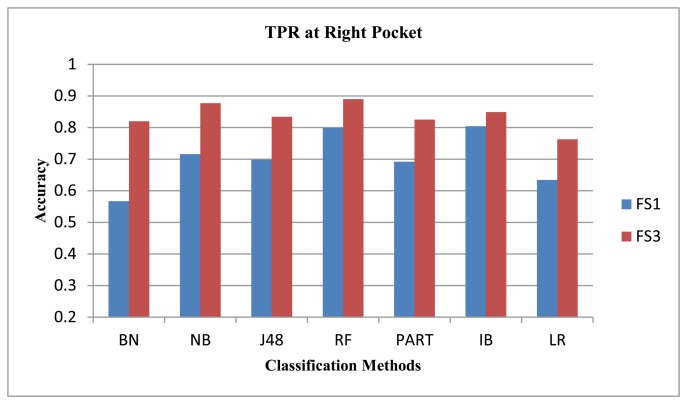
The magnetometer performance for the walking activity.

**Figure 7.**
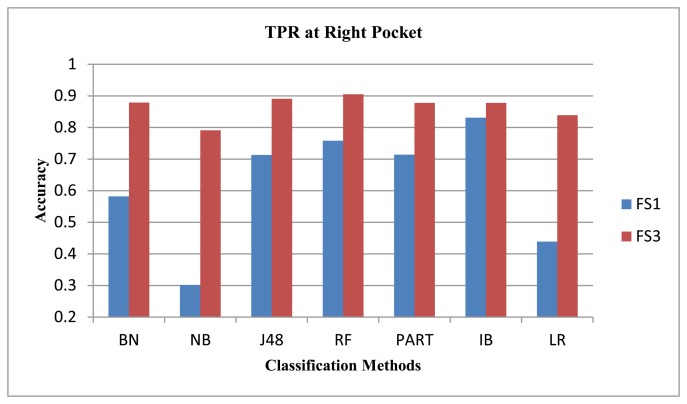
Magnetometer performance for the jogging activity.

We showed in our previous work [31] that the magnetometer performed poorly compared to the gyroscope and the accelerometer. We observed similar results here for our data set using FS1 only. We argued in our previous work that the magnetometer performance may improve if direction-insensitive features are used. In our earlier work, we evaluated the magnetometer using feature set FS1, where the ‘mean’ feature changes much if the direction changes. In this new study, we evaluate it using another feature set, FS3, comprised of variance, zero crossings and root mean square values. All these three features are less sensitive to the direction dependence in magnetometer. We show the performance for all classifiers, except LSM and NNGE, as they performed poorly with FS3. After evaluations, we see performance improvement for the walking and jogging activities, as shown in Figures 6 and 7, respectively. The average performance improvement for the walking and jogging activities using FS3 was 13% and 24%, respectively. For the biking, walking downstairs, sitting and standing activities, the performance remained almost the same. For walking upstairs, it was lower than that of FS1 by an average of 8%. We did these evaluations for the magnetometer, both with and without an extra magnitude dimension. The performance trends were similar in both cases, except for the walking downstairs activity, where we observe 5% improvement using FS3 with the extra magnitude dimension. Therefore, we only show the performance with extra magnitude. These results show that the magnetometer can be used in the activity recognition process if we select the correct feature set for it. Our current goal is not to improve the performance of the individual sensors; we leave it as future work to explore this sensor further.

### 6.4. Lessons Learned

We observe that the classification accuracy with the evaluated sensors depends on factors like the activities being recognized, the classification method, the body position and the feature set. Therefore, it is hard to make a generic statement on how these sensors would behave. However, their performance can be predicted in specific scenarios if we fix these other factors. Based on these evaluation results, we observe the following:

- For recognizing the walking downstairs and walking upstairs activities, the gyroscope performs better than accelerometer at the pocket and belt positions in most cases.
- For recognizing the sitting and standing activities, the accelerometer always performs better than the gyroscope at all five positions, and the gyroscope performs very poorly.
- For recognizing the biking, jogging and walking activity, the accelerometer performs slightly better than the gyroscope. However, the gyroscope also recognizes these activities with reasonable accuracy.
- The gyroscope and the accelerometer generally complement each other when used in combination if there is room for improvement in the overall performance. If one of these two sensors get high accuracy individually, then adding another sensor will not bring any improvement in the overall performance.
- The linear acceleration sensor follows the same trends as the accelerometer with very few exceptions. However, its performance is better than the accelerometer at the upper arm position and poorer at the belt and pocket position. Moreover, it performs poorly in differentiating between the sitting and standing activities, as the effect of gravity was removed.
- The magnetometer has the potential to be a candidate in the activity recognition process if we select the correct features for it, which are less sensitive to its direction dependence. Blindly combining different sensors should be avoided. Each sensor involved in the fusion should be individually evaluated offline for its contribution in the whole process.
- Making generic statements about the role of these sensors should be avoided when they are evaluated in a very specific scenario. Unlike the existing perception about the gyroscope, it can take the lead roles and perform reasonably well for recognizing some activities when used alone.

We showed these results for some specific, but commonly used, scenarios in the state-of-the-art studies. The results may differ if different classification methods, different features sets or body positions are used. Moreover, we kept the classification algorithm parameters at their default values. Changing these parameters may also lead to different results. We did not play with the optimizations of these algorithms, because then, there can be too many evaluation scenarios to handle. This can be explored as a future work. Moreover, we kept the orientation of the smartphone fixed, and it is yet to be investigated how the results will vary with orientation changes. Particularly, it is unknown to what extent the relative performance trends will change with varying phone orientation.

## 7. Conclusions and Future Work

We evaluated the activity recognition performance with four motion sensors using nine classifiers on five body positions with four feature sets. In the data collection experiments, seven physical activities were targeted. Our data set is publicly available with our data collection tool, which can be used for further studies based on this work.

Based on our evaluations, we show that both the accelerometer and the gyroscope are capable of taking the lead roles in the activity recognition process, depending on the type of activity being recognized, the body position, the classification method and the feature set being used. These two sensors take the lead in different situations. For example, the walking upstairs and walking downstairs activities are better recognized by the gyroscope in most of the situations. On the other hand, the standing and sitting activities are better recognized by the accelerometer. For the walking, biking and jogging activities, the accelerometer performs slightly better than the gyroscope. Moreover, their combination improves the overall TPR or at least keeps it equal to the maximum of their individual performances in almost all situations with very few exceptions. We evaluated the linear acceleration sensor, which should be less sensitive to the orientation changes. This sensor performed very similar to the accelerometer in comparison with the gyroscope. We also evaluate the magnetometer's role and show that it can recognize different activities in a better way if the correct features are extracted for it. Based on our evaluations, we conclude that it is difficult to make an exact generic statement about the role of these sensors in the activity recognition process for all situations. However, we can make statements about their roles in particular situations. These results can be used as the basis for implementing real-time activity recognition applications on smartphones and will help in making design decisions for when to combine these sensors.

This work can be further extended. For example, these results can be validated with more activities. It can also be validated on a different set of features. We used all the classification methods in their default settings, and therefore, the effect of different parameter settings can be explored. We kept the orientation for the smartphone fixed, so it will be interesting to see which of the evaluated sensors are more sensitive to orientation changes. Moreover, the feasibility of a magnetometer in activity recognition can be studied further.

## Figures and Tables

**Table 1.** Feature Sets.

Feature Set	Features in the Feature Set	Frequency or Time Domain
FS1	Mean, standard deviation	Time domain
FS2	Median, zero crossings, root means square	Time domain
FS3	Variance, zero crossings, root means square	Time domain
FS4	Sum of first five FFT coefficients, spectral energy	Frequency Domain

**Table 2.** Feature Complexities.

Feature Set	Associated Features	Computation Complexity	Storage Complexity	Suitability for Mobile Devices
FS1	Mean	very low	very low	yes
FS1	Standard Deviation	very low	very low	yes
FS2	Median	medium	very low	yes
FS2	Variance	very low	very low	yes
FS2, FS3	Zero crossings	very low	very low	yes
FS2, FS3	Root mean square value	very low	very low	yes
FS4	Sum off FFT coefficients	medium	low	moderate
FS4	Signal energy	medium	low	moderate

**Table 3.** Classification Methods.

Type of Classifier	WEKA-Version	Notation
Bayesian networks	Bayesian networks	BN
Naive Bayes	Naive Bayes	NB
Support vector machines	LibSVM	LSM
Logistic regression	Logistic regression	LR
K nearest neighbor	IB1 (KNN with K = 1)	IB
Rule-based classifiers	PART	PART
Rule-based classifiers	NNGE	NNGE
Decision trees	J48	J48
Decision trees	Random forest	RF

**Table 4.** Evaluation scenarios for the accelerometer and the gyroscope.

Scenarios	Feature Sets	Positions (Individual/Combined)	Classification (Personalized/Generalized)
A	FS1, FS2, FS4	Individual	Generalized
B	FS1, FS2, FS4	Combined (multiple positions)	Generalized
C	FS1	Individual	Personalized

**Table 5.** Leading role: Accelerometer *vs.* gyroscope for the walking downstairs activity.

Classifiers	Upper Arm	Wrist	Belt	Right Pocket
J48	*G(FS2)*/**A(FS4)**/E(FS1)	*G(FS2,4)*/E(FS1)	*G(FS2,4)*/**A(FS1)**	*G(FS1,2,4)*
RF	*G(FS1,2)*/**A(FS4)**	*G(FS1,2,4)*	*G(FS2,4)*/**A(FS1)**	*G(FS1,2,4)*
NNGE	*G(FS2)*/**A(FS4)/**E(FS1)	*G(FS2,4)*/E(FS1)	*G(FS2,4)*/**A(FS1)**	*G(FS1,2)*/E(FS4)
PART	*G(FS1,2)*/**A(FS4)**	*G(FS2,4)*/E(FS1)	*G(FS2,4)*/**A(FS1)**	*G(FS1,2)/*E(FS4)
IB	*G(FS1,2)*/**A(FS4)**	*G(FS2,4)*/E(FS1)	*G(FS2,4)*/**A(FS1)**	*G(FS1,2,4)*
BN	*G(FS1,2)*/E(FS4)	**A(FS1,2,4)**	*G(FS2)*/**A(FS1,4)**	*G(FS1,2*)/E(FS4)
NB	*G(FS2,4)*/E(FS1)	*G(FS4)*/**A(FS1,2)**	*G(FS2)*/**A(FS4)**/E(FS1)	*G(FS1,2*)/**A(FS4)**
LSM	E(FS1,2)	E(FS1,2)	**A(FS1,2)**	*G(FS2*)/**A(FS1)**
LR	*G(FS4)*/E(FS1,2)	*G*(*FS4*)/**A(FS2)**/E(FS1)	*G*(*FS4*)/**A(FS1)**/E(FS2)	*G(FS2*)/**A(FS4)**/E(FS1)

**Table 6.** Leading role: Accelerometer *vs.* gyroscope for the walking upstairs activity.

Classifiers	Upper arm	Wrist	Belt	Right pocket
J48	**A(FS4)/**E(FS1,2)	*G(FS4)*/E(FS1,2)	*G(FS2,4)*/**A(FS1**)	*G(FS1,2,4)*
RF	**A(FS2,4**)/E(FS1)	*G(FS2,4)*/**A(FS1)**	*G(FS4)*/**A(FS1**)/E(FS2)	*G(FS1,2,4)*
NNGE	**A(FS4**)/E(FS1,2)	*G(FS4)*/E(FS1,2)	*G(FS2,4)*/**A(FS1)**	*G(FS1,2)*/E(FS4)
PART	*G(FS1,2)*/E(FS4)	*G(FS4)*/E(FS1,2)	*G(FS2,4)*/**A(FS1**)	*G(FS1,2,4)*
IB	E(FS1,2,4)	*G(FS2,4)*/**A(FS1)**	*G(FS2,4)*/E(FS1)	*G(FS1,2,4)*
BN	**A(FS1,2,4)**	*G(FS2)*/**A(FS1, 4)**	*G(FS2,4)*/**A(FS1)**	*G(FS2*)/**A(FS1,4)**
NB	**A(FS1,2,4)**	**A(FS2,4)**/E(FS1)	**A(FS1,2,4)**	*G(FS2*)/**A(FS1,4)**
LSM	**A(FS1,2)**	**A(FS1,2)**	**A(FS1,2)**	*G(FS2*)/**A(FS1)**
LR	**A(FS2,4)**/E(FS1)	**A(FS1,2**)/E(FS4)	*G(FS2,4)*/**A(FS1)**	*G(FS2*)/**A(FS1,4)**

**Table 7.** Leading role: Accelerometer *vs.* gyroscope for the walking activity.

Classifiers	Upper Arm	Wrist	Belt	Right Pocket
J48	*G(FS4)*/**A(FS1,2)**	**A(FS1)/**E(FS2,4)	*G(FS4)*/**A(FS1)/**E(FS2)	**A(FS1)**/E(FS2,4)
RF	*G(FS4)*/**A(FS1)**/E(FS2)	**A(FS1)/**E(FS2,4)	*G(FS4)*/**A(FS1)/**E(FS2)	**A(FS1)**/E(FS2,4)
NNGE	**A(FS1)/**E(FS2,4)	**A(FS1,2,4)**	*G(FS2,4)*/E(FS1)	*G(FS4)*/**A(FS1,2)**
PART	**A(FS1)/**E(FS2,4)	*G(FS4)*/**A(FS1)/**E(FS2)	*G(FS4)*/**A(FS1)/**E(FS2)	*G(FS4)/***A(FS1)**/E(FS2)
IB	**A(FS1)/**E(FS2,4)	**A(FS1,2,4)**	*G(FS4)*/**A(FS1)**/E(FS2)	E(FS1,2,4)
BN	**A(FS1,2,4)**	*G(FS4)*/**A(FS1)**/E(FS2)	*G(FS1.4)*/E(FS2)	**A(FS1,2,4)**
NB	**A(FS1,2,4)**	*G(FS2,4)*/E(FS1)	*G(FS1,2,4)*	**A(FS1,2,4)**
LSM	**A(FS1,2)**	**A(FS1)**/E(FS2)	**A(FS1,2)**	**A(FS1,2))**
LR	*G(FS4)*/**A(FS1)**/E(FS2)	*G(FS2,4)*/**A(FS1)**	*G(FS1,2,4)*	**A(FS2,4)/**E(FS1)

**Table 8.** Leading role: Linear acceleration sensor *vs.* accelerometer using nine classifiers.

Activities	Upper Arm	Wrist	Belt	Right Pocket
Walking	*LA(FS2,4)/*E(FS1)	E(FS1,2,4)	**A(FS2,4)/***LA(FS1)*	**A(FS1,2,4)**
Standing	**A(FS1,2,4)**	**A(FS1,2,4)**	**A(FS1,2,4)**	**A(FS1,2,4)**
Jogging	E(FS1,2,4)	E(FS1,2,4)	E(FS1,2,4)	E(FS1,2,4)
Sitting	**A(FS1,2,4)**	**A(FS1,2,4)**	**A(FS1,2,4)**	**A(FS1,2,4)**
Biking	**A(FS1)/**LA(FS2,4)	**A(FS1)/**E(FS2,4)	**A(FS1)/**E(FS2,4)	**A(FS1,2,4)**
Walking upstairs	*LA(FS1,2,4)*	**A(FS1,2)/***LA(FS4)*	**A(FS1,2,4)**	**A(FS1,2,4)**
Walking downstairs	*LA(FS1,2,4)*	**A(FS1,2)/***LA(FS4)*	**A(FS1,2,4)**	**A(FS1,2,4)**

**Table 9.** Evaluation scenarios for the linear acceleration sensor.

Scenarios	Feature Sets	Positions (Individual/Combined)	Classification (Personalized/Generalized)
D	FS1, FS2, FS4	Individual	Generalized
E	FS1, FS2, FS4	Combined (multiple positions)	Generalized

**Figure A.3.**
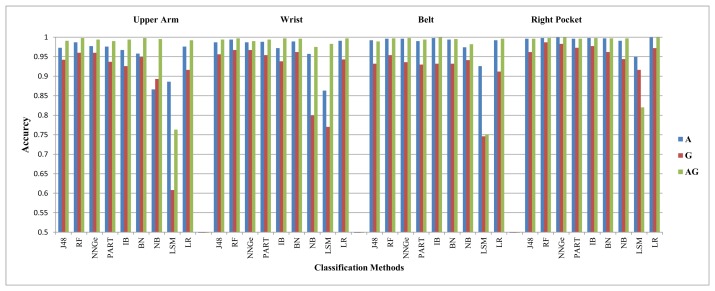
Recognition performance for the biking activity at all four positions using FS2.

**Figure A.5.**
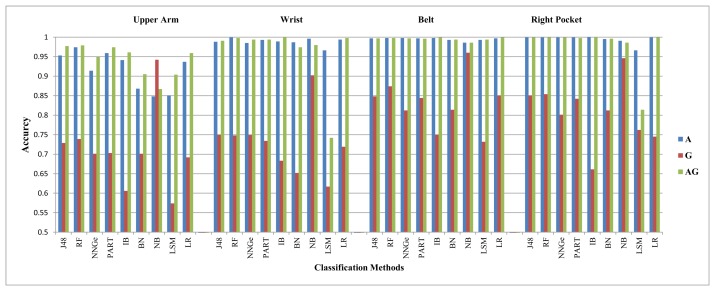
Recognition performance for the sitting activity at all four positions using FS2.

**Figure A.7.**
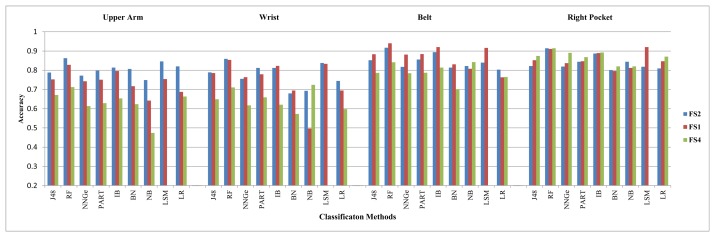
Accelerometer true positive rate (TPR) for the walking upstairs activity for all four positions and three feature sets.

**Figure A.9.**
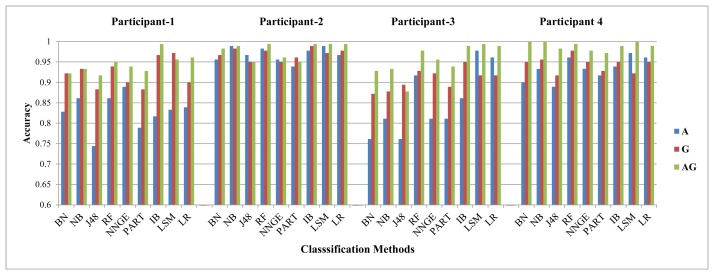
Recognition performance for the walking upstairs activity in Scenario C.

## Appendix

In this Appendix, we show some of the performance graphs and tables. Each cell in Tables A.1, A.2, A.3–A.4 are represented by the following equation:

(2) cell value=G−A

If the value in a table's cell is positive, this means that the gyroscope (*G*) performed better than the accelerometer (*A*) by that value and *vice versa*.

The values in Tables A.5, A.6, A.7–A.8 are represented by the following equation:

(3) cell value=AG−max(A,G)

If the value in a table's cell is positive, this means that the combination of the gyroscope and the accelerometer (*AG*) performed better than the maximum of the individual performances of the accelerometer (*A*) and the gyroscope (*G*) by that value and *vice versa*.

**Table A.1.** Performance difference (gyroscope (G)—accelerometer (A)) for the walking downstairs activity in Scenario A.

Classifiers	%Difference at Upper arm			% Difference at Wrist			% Difference at Belt			%Difference at Right Pocket		
	FS1	FS2	FS4	FS1	FS2	FS4	FS1	FS2	FS4	FS1	FS2	FS4
J48	−0.4	3.1	−4.0	−0.6	2.1	2.2	−8.2	4.4	4.2	5.0	8.3	4.2
RF	4.0	5.8	−2.0	2.2	3.4	6.4	−7.4	4.9	8.5	5.3	11.4	4.8
NNGe	−0.8	3.9	−5.6	−0.3	3.6	2.9	−10.0	2.9	2.8	6.7	10.5	1.0
PART	4.4	5.2	−3.6	1.8	4.1	4.2	−9.0	2.7	8.7	4.4	9.8	0.9
IB	3.0	5.0	−5.0	1.6	5.1	7.9	−6.1	3.0	5.5	7.6	8.7	4.9
BN	14.3	10.2	−1.6	−6.9	−6.2	−5.1	−16.2	4.9	−7.7	16.4	12.8	1.2
NB	1.7	10.4	16.7	−10.2	−12.2	5.4	−0.6	5.4	−6.4	4.9	13.5	−15.3
LSM	−0.2	−1.9	NA	1.0	0.1	NA	−30.6	−7.7	NA	−9.7	5.3	NA
LR	1.5	0.1	2.3	−0.4	−2.1	10.3	−11.9	1.7	18.6	0.8	11.5	−14.3

**Table A.2.** Performance difference (gyroscope (G)—accelerometer (A)) for the walking upstairs activity in Scenario A.

Classifiers	%Difference at Upper arm			% Difference at Wrist			% Difference at Belt			%Difference at Right Pocket		
	FS1	FS2	FS4	FS1	FS2	FS4	FS1	FS2	FS4	FS1	FS2	FS4
J48	1.9	0.9	−3.2	0.6	3.5	3.5	−3.5	2.3	8.6	3.0	12.1	3.3
RF	−1.0	−2.6	−2.5	−2.5	0.3	3.6	−4.5	1.0	8.3	2.0	6.1	2.8
NNGe	−1.2	−1.3	−3.7	−1.2	5.1	3.6	−3.4	9.0	8.9	6.1	14.2	1.9
PART	3.2	2.0	1.0	0.3	0.5	3.2	−5.5	4.2	10.4	3.0	10.1	4.6
IB	0.4	1.7	−1.0	−2.6	1.4	5.2	−1.5	3.6	7.7	6.6	8.8	4.4
BN	−13.6	−13.8	−20.5	−5.6	8.5	−11.0	−15.8	5.7	3.2	−8.1	13.9	−15.9
NB	−17.5	−18.1	−23.6	1.9	−6.1	−40.5	−30.6	−6.8	−31.1	−18.1	6.6	−22.0
LSM	−8.7	−9.5	NA	−8.5	−4.1	NA	−20.0	−5.1	NA	−8.7	11.4	NA
LR	1.2	−7.6	−7.6	−2.6	−2.2	−0.7	−12.0	4.3	6.0	−11.1	13.7	−2.2

**Table A.3.** Performance difference (gyroscope—accelerometer) for the walking activity in Scenario A.

Classifiers	%Difference at Upper Arm			%Difference at Wrist			% Difference at Belt			%Difference at Right Pocket		
	FS1	FS2	FS4	FS1	FS2	FS4	FS1	FS2	FS4	FS1	FS2	FS4
J48	−3.2	−2.1	2.3	−8.6	0.7	1.1	−3.5	0.9	3.9	−2.9	0.1	1.7
RF	−2.8	−1.0	2.1	−6.9	−0.3	0.8	−3.3	−1.1	3.5	−2.3	−1.7	0.6
NNGe	−4.6	−1.8	−0.1	−13.2	−4.6	−3.2	−1.5	3.4	3.2	−2.7	−2.5	2.3
PART	−5.1	1.1	−0.4	−8.3	0.9	5.5	−3.3	0.0	5.4	−2.2	−1.1	2.2
IB	−2.0	−1.1	0.2	−6.3	−2.1	−3.1	−3.2	−0.4	2.0	−0.5	−0.1	1.4
BN	−15.0	−9.6	−16.6	−3.5	−0.4	2.3	2.4	0.8	2.4	−7.1	−5.8	−3.9
NB	−8.4	−4.5	−38.2	−0.2	7.6	39.4	44.5	26.3	56.7	−7.3	−9.4	−9.0
LSM	−8.3	−2.9	NA	−16.1	0.9	NA	−12	−5.0	NA	−3.6	−5.0	NA
LR	−6.8	−0.9	10.4	−11.6	5.0	17.9	14.4	7.6	37.1	−0.6	−2.9	−23.5

**Table A.4.** Performance difference (gyroscope—accelerometer) for the walking, walking downstairs and walking upstairs activities in Scenario B.

Classifiers	% Difference for Walking			% Difference for Walking Downstairs			% Difference for Walking Dpstairs		
	FS1	FS2	FS4	FS1	FS2	FS4	FS1	FS2	FS4
J48	−7.4	−2.1	1.2	2.0	5.3	3.0	1.7	2.8	−0.6
RF	−5.6	−2.3	−0.2	3.8	5.8	5.1	−2.9	1.3	1.6
PART	−11.6	−2.7	1.2	1.1	5.0	3.0	−6.4	3.7	0.1
IB	−4.6	−2.2	−1.8	3.2	5.3	0.3	−0.1	2.1	2.1
BN	−9.0	1.3	0.2	3.2	6.8	10.0	−2.8	5.5	−20.5
NB	38.0	10.8	19.4	−12.3	7.3	−6.2	−23.2	3.4	−41.5
LSM	−19.2	−5.9	NA	−1.4	1.1	NA	−17.1	−2.5	NA
LR	7.9	27.2	−2.7	1.1	18.7	4.0	−12.8	22.7	−12.2

**Table A.5.** Performance difference (AG—max(A,G)) for the walking downstairs activity in Scenario A.

Classifiers	%Difference at Upper arm			% Difference at Wrist			%Difference at Belt			%Difference at Right Pocket		
	FS1	FS2	FS4	FS1	FS2	FS4	FS1	FS2	FS4	FS1	FS2	FS4
J48	14	5.9	11.9	9.2	6.1	11.1	7.4	4.6	8.0	9.2	7.6	8.1
RF	10.9	5.4	12.5	8.4	5.2	9.9	8.8	5.6	7.6	7.6	4.5	7.8
NNGe	14.3	6.8	12.4	10.6	7.3	12.2	10.3	7.8	9.6	12.4	7.2	11.6
PART	11.3	5.1	11.8	9.0	7.0	9.7	7.6	5.6	6.2	8.9	4.5	9.0
IB	13.0	7.4	12.2	9.6	6.3	10.3	8.6	7.9	11.1	5.3	3.3	9.4
BN	10.6	9.1	12.8	20.4	9.7	11.7	18.3	10.9	19.1	16.0	10.0	22.0
NB	21.3	10.5	−4.5	19.3	9.2	6.7	13.6	13.8	8.0	32.5	11.0	27
LSM	18.2	7.2	NA	12.8	5.5	NA	12.9	2.2	NA	9.4	2.3	NA
LR	15.9	11.8	17.5	12.7	12.0	16.9	23.3	12.4	13.1	20.8	10.5	9.8

**Table A.6.** Performance difference (AG − max(A,G)) for the walking upstairs activity in Scenario A.

Classifiers	%Difference at Upper arm			% Difference at Wrist			% Difference at Belt			%Difference at Right Pocket		
	FS1	FS2	FS4	FS1	FS2	FS4	FS1	FS2	FS4	FS1	FS2	FS4
J48	12.5	8.0	12.0	10.0	6.3	9.8	6.5	5.4	5.1	7.4	2.0	8.1
RF	10.4	7.9	16.5	7.8	8.2	12.3	4.3	4.1	4.2	5.3	1.2	7.8
NNGe	10.8	11.0	10.8	8.0	3.8	2.9	4.8	0.8	4.5	4.4	−0.7	11.6
PART	13.1	7.0	17.6	11.7	8.0	11.1	7.0	5.4	3.9	7.8	2.3	9.0
IB	15.2	12.6	19.7	12.4	11.8	16.4	6.8	5.4	7.8	4.3	1.9	9.4
BN	10.7	9.4	11.4	15.6	5.5	7.9	6.9	5.6	13.9	15.7	3.9	22.0
NB	6.7	9.2	12.5	18.4	10.0	−13.3	−7.5	5.4	−2.5	11.9	6.2	27.0
LSM	10.5	6.6	NA	7.1	6.8	NA	3.2	−0.2	NA	7.1	−3.1	NA
LR	12.9	7.9	6.8	14.3	13	12.1	11.8	8.7	7.7	12.5	3.3	9.8

**Table A.7.** Performance difference (AG—max(A,G)) for the walking activity in Scenario A.

Classifiers	%Difference at Upper arm			% Difference at Wrist			% Difference at Belt			%Difference at Right Pocket		
	FS1	FS2	FS4	FS1	FS2	FS4	FS1	FS2	FS4	FS1	FS2	FS4
J48	4.1	7.4	9.0	1.7	3.8	4.1	3.7	2.3	3.6	1.7	2.9	1.3
RF	3.9	4.6	7.4	1.4	2.5	4.0	1.4	1.4	2.3	0.8	0.3	1.5
NNGe	3.9	4.5	9.8	−3.6	−2.3	4.7	0.8	−1.7	2.2	−0.2	−0.2	0.9
PART	6.0	7.4	7.3	2.0	4.6	3.9	3.6	3.6	2.5	2.9	1.4	2.4
IB	6.3	5.5	12.6	1.4	2.9	6.0	2.3	1.9	3.4	0.9	1.2	2.4
BN	3.6	4.1	5.2	7.5	4.2	11.4	7.7	4.4	9.0	1.7	2.5	2.0
NB	7.0	5.0	−1.7	12.3	9.4	3.7	−7.3	−5.1	−15.4	3.5	0.9	−4.6
LSM	2.0	5.2	NA	1.6	4.8	NA	2.5	0.9	NA	0.7	−1.9	NA
LR	4.6	10.8	6.3	8.7	10.6	3.2	24.7	7.2	9.5	7.9	2.3	2.2

**Table A.8.** Performance difference (AG—max(A,G)) for the walking, walking downstairs and walking upstairs activities in Scenario B.

Classifiers	% Difference for Walking			%Difference for Walking Downstairs			%Difference for Walking Upstairs		
	FS1	FS2	FS4	FS1	FS2	FS4	FS1	FS2	FS4
J48	2.0	3.7	6.1	10.9	6.0	10.6	12.4	7.2	12.0
RF	1.7	2.0	5.5	10.9	6.8	12.0	9.7	7.9	12.8
PART	−1.1	4.8	7.3	11.4	5.9	11.2	6.2	7.3	12.3
IB	2.5	3.3	8.5	11.4	6.8	16.1	13.4	11.4	17.2
BN	10.1	5.7	14.7	19.2	12.3	5.5	20.9	11.3	11.2
NB	10.1	−8.2	16.0	16.0	15.2	−5.0	−6.8	14.3	−22.2
LSM	1.2	3.5	NA	16.2	5.7	NA	10.0	7.4	NA
LR	14.2	−7.5	13.8	16.7	1.0	14.4	12.6	−1.5	3.0
